# Statistical investigation of simulated fed intestinal media composition on the equilibrium solubility of oral drugs

**DOI:** 10.1016/j.ejps.2016.12.008

**Published:** 2017-03-01

**Authors:** Zhou Zhou, Claire Dunn, Ibrahim Khadra, Clive G. Wilson, Gavin W. Halbert

**Affiliations:** Strathclyde Institute of Pharmacy and Biomedical Sciences, University of Strathclyde, 161 Cathedral Street, Glasgow, G4 0RE, United Kingdom

**Keywords:** BCS, Biopharmaceutics Classification System, DoE, Design of Experiment, FASSIF, Fasted Simulated Intestinal Fluid, FESSIF, Fed Simulated Intestinal Fluid, IVIVC, In vitro In vivo correlation, Fed simulated intestinal fluid, Design of experiment, Biopharmaceutics classification system, Equilibrium solubility

## Abstract

Gastrointestinal fluid is a complex milieu and it is recognised that gut drug solubility is different to that observed in simple aqueous buffers. Simulated gastrointestinal media have been developed covering fasted and fed states to facilitate in vitro prediction of gut solubility and product dissolution. However, the combination of bile salts, phospholipids, fatty acids and proteins in an aqueous buffered system creates multiple phases and drug solubility is therefore a complex interaction between these components, which may create unique environments for each API. The impact on solubility can be assessed through a statistical design of experiment (DoE) approach, to determine the influence and relationships between factors. In this paper DoE has been applied to fed simulated gastrointestinal media consisting of eight components (pH, bile salt, lecithin, sodium oleate, monoglyceride, buffer, salt and pancreatin) using a two level D-optimal design with forty-four duplicate measurements and four centre points. The equilibrium solubility of a range of poorly soluble acidic (indomethacin, ibuprofen, phenytoin, valsartan, zafirlukast), basic (aprepitant, carvedilol, tadalafil, bromocriptine) and neutral (fenofibrate, felodipine, probucol, itraconazole) drugs was investigated. Results indicate that the DoE provides equilibrium solubility values that are comparable to literature results for other simulated fed gastrointestinal media systems or human intestinal fluid samples. For acidic drugs the influence of pH predominates but other significant factors related to oleate and bile salt or interactions between them are present. For basic drugs pH, oleate and bile salt have equal significance along with interactions between pH and oleate and lecithin and oleate. Neutral drugs show diverse effects of the media components particularly with regard to oleate, bile salt, pH and lecithin but the presence of monoglyceride, pancreatin and buffer have significant but smaller effects on solubility. There are fourteen significant interactions between factors mainly related to the surfactant components and pH, indicating that the solubility of neutral drugs in fed simulated media is complex. The results also indicate that the equilibrium solubility of each drug can exhibit individualistic behaviour associated with the drug's chemical structure, physicochemical properties and interaction with media components. The utility of DoE for fed simulated media has been demonstrated providing equilibrium solubility values comparable with similar in vitro systems whilst also providing greater information on the influence of media factors and their interactions. The determination of a drug's gastrointestinal solubility envelope provides useful limits that can potentially be applied to in silico modelling and in vivo experiments.

## Graphical abstract

**Figure:**
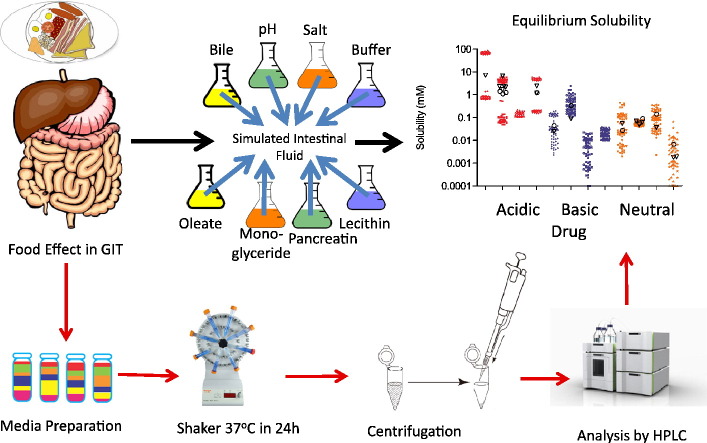

## 1 Introduction

The current trend in drug discovery towards molecules with a higher molecular weight and increased lipophilicity continues to result in a greater number of drug candidates with decreasing aqueous solubility (Sugano et al., 2007), (Lipinski, 2000). Aqueous solubility is a key parameter influencing biological activity (Stegemann et al., 2007), formulation (Pouton, 2006) and in vitro and in vivo biopharmaceutical performance (Lipinski, 2000). Aqueous solubility may be determined in vitro using a number of experimental techniques (Sugano et al., 2007). Intrinsic solubility is a measure of the neutral (non-ionised) molecule's maximum solubility (Yalkowsky, 1999) in aqueous solution, whilst equilibrium solubility includes both un-ionised and ionised forms using a defined aqueous system (pH and presence of other salts) and employing the drug's most stable solid form in contact with the solution. Either value can be measured using classical shake-flask methods (Dittert et al., 1964) where an excess of solid drug is mixed with a buffered solution phase until equilibrium is achieved. During oral administration and absorption, an equilibrium concentration is unlikely to exist due to the competing processes of dissolution and absorption although equilibrium aqueous solubility is demonstrably still a key parameter controlling rate and extent of absorption (Sugano and Terada, 2015). This is recognised in the Biopharmaceutics Classification System where drugs are allocated to categories based on solubility with respect to dose either high or low and gastrointestinal permeability (Amidon et al., 1995). Low solubility drugs present problems during formulation and development (Butler and Dressman, 2010) and in order to avoid solubility related failures during drug discovery, an early and comprehensive assessment of a drug's solubility is essential (Bergstrom et al., 2014).

Peroral drug administration is the most convenient and popular method for drug therapy covering a range of diseases and applications from acute through to chronic dosing. The normal function of the gastrointestinal tract is to provide efficient nutrition from a range of food matrices, coupled with excretion of metabolic waste products. This is accomplished by a dynamic, responsive secretion of fluids, and appropriate muscular activity to mix food, extract nutrients with the residues being pushed forward. It is appreciated therefore that the dynamic and complex physiology of the gastrointestinal tract influences drug absorption (Varum et al., 2013). Two major features of the gut are the inherent physicochemical conditions within the tract which vary with position along the tract (Bergstrom et al., 2014, Mudie et al., 2010) and the effect of ingested food (Yasuji et al., 2012) on these conditions, both of which exhibit intra- and inter-subject variability. Simple aqueous drug solubility therefore cannot reflect gastrointestinal solubility (Dressman et al., 2007) and in order to improve this determination in vitro, either sampled human fluids can be employed (Augustijns et al., 2014) or simulated gastrointestinal media prepared (Vertzoni et al., 2004). Human gastrointestinal fluids are expensive and problematical to sample, variable in composition (Bergstrom et al., 2014, Riethorst et al., 2016), unstable in air and therefore not an ideal material for in vitro experimental studies. Simulated gastrointestinal media are more easily prepared and two initial recipes simulating the fed state were published in 1998 (Dressman et al., 1998, Galia et al., 1998) see Table 1. Several adaptations have been investigated, for example changing the buffer to citrate (Vertzoni et al., 2004) or maleate (Jantratid et al., 2008a) and modification of the bile salt and lecithin concentration and ratio plus the inclusion of additional components such as monoglyceride or fatty acid (Jantratid et al., 2008b, Kleberg et al., 2010). However, a fixed composition simulated media reflects a single physicochemical state usually based around the average of measured parameters. As already discussed, gastrointestinal fluid composition is highly variable (Riethorst et al., 2016) and the situation is further confounded by changes in fluid composition as the mass passes along the small intestine (Bergstrom et al., 2014).

In order to investigate the influence of simulated fasted gastrointestinal media composition on the equilibrium solubility of twelve test drugs (four acidic, four basic and four neutral), we have employed a design of experiment (DoE) (Myers et al., 2009) type approach using published literature composition values for fasted gastrointestinal fluid (Khadra et al., 2015). This study illustrated that the DoE approach was feasible, simulated the inherent solubility variability associated with fasted gastrointestinal fluid and identified the key media components controlling solubility. For acidic drugs, pH was the major factor, whilst for basic and neutral compounds a combination of pH and the concentrations of fatty acid, bile salt and lecithin were important. The DoE also highlighted interactions between media components, for example pH and fatty acid, an interdependence that would otherwise have been undetected and also identified drugs where solubility behaviour was unusual or influenced by media components or interactions.

**Fig. 1:**
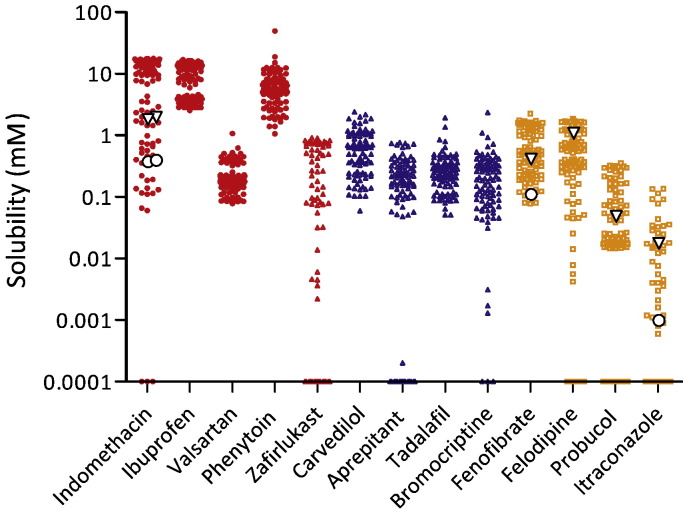
Design of experiment equilibrium solubility measurements. Equilibrium solubility measurements for each drug (acidic red coloured points; basic blue coloured points; neutral yellow coloured points) based on media compositions detailed in Table 2. ○ reported solubility values for individual drugs in FeSSIF, ∇ reported solubility values for individual drugs in fed HIF, all values from (Augustijns et al., 2014).

In this paper we have extended the DoE approach (Khadra et al., 2015) to simulated fed gastrointestinal media using the same components at higher concentrations and with the addition of monoglyceride as an additional fed media component, Table 1 (Jantratid et al., 2008a, Kleberg et al., 2010). The lower and upper concentration values of the experiment are presented in Table 2 and are based on published measured fed intestinal fluid ranges as reviewed by Bergstrom and colleagues (Bergstrom et al., 2014) (see Figs. 1, 6, 9 and 10) and typical concentrations employed by previously published simulated fed media, see Table 1. The addition of a factor to a fractional factorial DoE would double the number of required test conditions if the power of the experiment was to remain constant. In order to limit the number of conditions tested, the experimental design has been changed to a D-optimal design, which accommodates the same number of factors with less experiments. The D-optimal design provides an increased resolution of the main effects but with a reduced resolution of two way interactions. Finally, the HPLC method has been simplified to a single method accommodating all tested drugs.

## 2 Materials and methods

### 2.1 Materials

Hydrochloric acid (HCl), potassium hydroxide (KOH), acetic acid, sodium taurocholate, lecithin S PC (phosphatidylcholine from Soybean “98%”) from Lipoid, Germany and Pancreatin from porcine sources, monosodium phosphate (NaH_2_PO_4_), sodium chloride (NaCl), chloroform, fenofibrate, and indomethacin were purchased from Sigma-Aldrich, Poole, Dorset UK. The active pharmaceutical ingredients aprepitant, carvedilol, felodipine, probucol, tadalafil and zafirlukast were kindly provided through OrBiTo (see Acknowledgements) by Dr. R. Holm Head of Preformulation, Lundbeck, Denmark. Itraconazole, bromocriptine, valsartan and phenytoin were purchased from Sigma, Poole, Dorset, UK. Sodium oleate was obtained from BDH Chemical Ltd. Poole England. All water used was ultrapure Milli-Q water. The analytical solvents methanol and acetonitrile were of HPLC grade (VWR, UK). Other materials used in this study included trifluoroacetic acid (Merck Schuchardt OHG, Germany) and ammonium acetate (Merck, Germany).

### 2.2 Design of experiment and data analysis

A D-Optimal DoE with 8 factors (either a component concentration or a system parameter such as pH) and 2 levels was constructed and analysed using MODDE (Umetrics) with the design selected using G-efficiency, which required 92 different experiments (44 conditions each measured in duplicate and 4 repeating centre points). Two assumptions were made when designing and analysing the DoE. First, only main effects and 2-way interactions (quadratic terms) are included in the model, and 3-way (or more) interactions were not determined. Secondly, it was proposed that the main effect can be positive (+) or negative (−), but when it is involved in interaction, the conclusion will be considered with the interactions (±).

### 2.3 Equilibrium solubility measurements

#### 2.3.1 Preparation of lipid stock solutions

Sodium taurocholate, lecithin or monoglyceride were weighed into a flask and 1 to 2 mL of chloroform added and mixed to dissolve all the solid material. Chloroform was removed in a stream of nitrogen gas to ensure a dry film was produced. Water (3 mL) was added to reconstitute the dried film, stirred to prepare a homogeneous mixture and transferred to a 5 mL volumetric flask and made to volume with water.

#### 2.3.2 Preparation of aqueous stock solutions

Salt Stock Solution: Sodium chloride (4.45 g) was weighed into a 25 mL volumetric flask, dissolved and made up to volume with water.

Buffer Stock Solution: Maleic acid (5.05 g) was weighed into a 50 mL volumetric flask in duplicate, dissolved in water 40 mL approx., the pH of each flask adjusted to 5 (Maleic buffer A) or 7 (Maleic buffer B) using 0.5 M HCl or 0.5 M KOH and made up to volume with water.

Sodium Oleate Stock Solution: Sodium oleate (3.81 g) was weighed into a 50 mL volumetric flask, dissolved in water under gentle heat and made to final volume. Solution was then kept at 50 °C to aid solubilisation.

Pancreatin Stock Solution: Pancreatin (2.86 g) was weighed in to a 20 mL volumetric flask, dissolved and made up to volume with water.

#### 2.3.3 Preparation of measurement solutions

The concentration of each stock solution was designed to be 15 times greater than the high concentration value required for the DoE, with the exception of sodium oleate where only a 5 times concentrate was possible. The stock solutions lipid and aqueous were combined in the preparation of the final DOE experimental solutions to provide the 44 conditions required by the model.

#### 2.3.4 Determination of equilibrium solubility

A weight of powdered drug greater than its estimated solubility (calculated from literature solubility values in FESSIF or HIF if available) was added to each 15 mL centrifuge tube (92 in total). The required amount of each stock solution (section above) and water was added to each tube to provide a final volume of 4 mL and the pH was adjusted to 5 or 7 using 0.5 M HCl or 0.5 M KOH. Tubes were shaken for 1 h at room temperature and examined for the presence of solid drug. If no solid drug was seen a further quantity of solid drug was added and above steps repeated. Tubes were then placed in an orbital shaker and incubated for 24 h at 37 °C and 240 rpm. Following incubation the pH of the tube was measured. Tubes were then centrifuged (10,000 rpm, 15 min) and 500 μL of supernatant was removed for solubilised drug concentration determination by HPLC. The supernatant was measured directly and not treated any further.

### 2.4 HPLC concentration measurement

HPLC was performed using an Agilent Technologies 1260 Series Liquid Chromatography system controlled by Clarity Chromatography software. Mobile Phase: A: 10 mM Ammonium formate pH 3.0 in water; B: 10 mM Ammonium formate pH 3.0 in MeCN/H2O (9:1 v/v), Flow rate 1.5 mL/min, Gradient: Time 0, 70%A:30%B, 3 min 0%A:100%B, 4 min 0%A:100%B, 4.5 min 70%A:30%B total run time 10 min; Column: ACE 3 μm C18,: 50 × 3.0 mm, Column Temperature: 60 °C, Injection volume: 10 μL, Detection: 214 nm.

## 3 Results and discussion

### 3.1 Equilibrium solubility measurements

The results for the individual equilibrium solubility measurements in each DoE experiment are presented in Fig. 1. For each drug variability is evident, which in some cases is up to three orders of magnitude. In addition, for several drugs very low solubility determinations were recorded for multiple media recipes. Literature equilibrium solubility values (where available) (Augustijns et al., 2014) in either HIF or fed simulated gastrointestinal media are superimposed on Fig. 1 and lie within the DoE values. It is interesting to note that although all the values lie within the solubility range measured, the HIF values are at the higher end of the range compared to the simulated media values. Overall the range of solubility values reported in Fig. 1 are higher than those for a fasted DoE (Khadra et al., 2015) an outcome that is in agreement with literature results for solubility differences between the fasted and fed states (Augustijns et al., 2014, Bevernage et al., 2010, Clarysse et al., 2011). The variability in solubility is drug dependent and mirrors literature variability values; for example, felodipine shows a greater variability than fenofibrate (Augustijns et al., 2014). However, the DoE may overestimate variability due to the statistical analysis of factor combinations that are not biorelevant. In addition the phase behaviour and emulsion homogeneity of each DoE point was not assessed and this also has the potential to influence solubility. The effect of the two level DoE, especially with respect to pH and influence on acidic drug solubility is not as evident as in a published fasted DoE (Khadra et al., 2015). The measured equilibrium solubility values indicate that the DoE covered the appropriate solubility space and mirrored the variability previously determined using alternative media systems.

### 3.2 Solubility influence of individual DoE factors

**Fig. 2:**
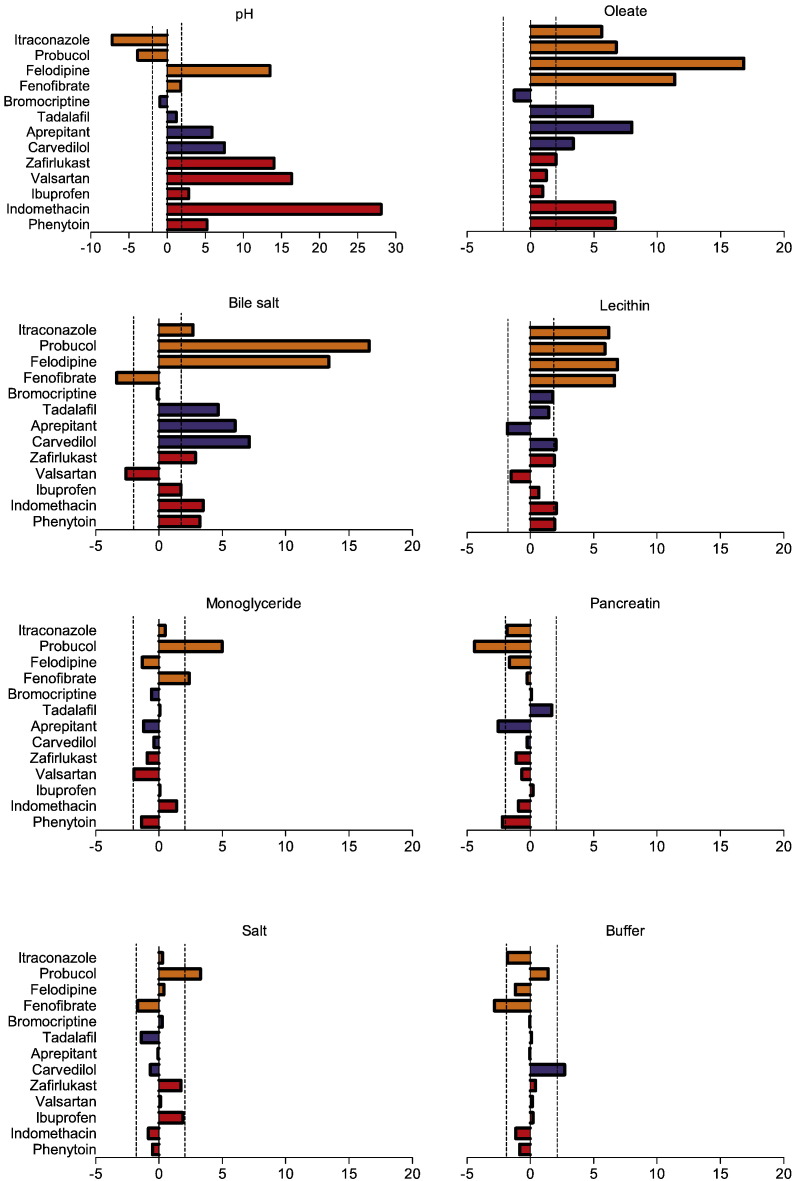
Standardised effect values for individual doe factors on equilibrium solubility. DoE standardised effect values (x-axis) for individual factors (as listed in figure titles) on equilibrium solubility. Vertical hatched black lines indicate statistical significance (*P* < 0.05), bar direction indicates direction of effect, to the right of 0 on x-axis is a positive effect on solubility, bar length indicates the magnitude of the effect. Acidic drugs red coloured bars; Basic blue coloured bars; Neutral yellow coloured bars.

**Fig. 3:**
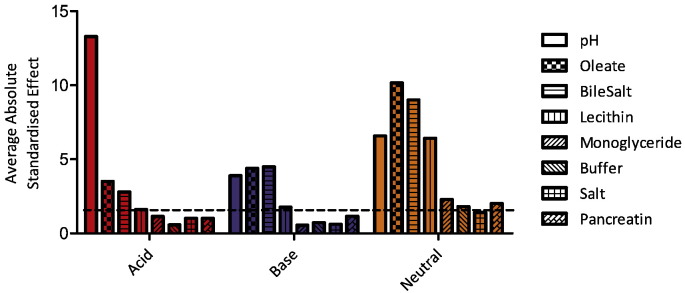
Average absolute standardised effect values grouped by drug category. Average value of the absolute standardised effect for each factor grouped by drug category, note that this removes direction of effect information. Horizontal hatched black line indicates statistical significance (*P* < 0.05). Acidic drugs red coloured bars; Basic blue coloured bars; Neutral yellow coloured bars.

The individual media components standardised effect on the measured equilibrium solubility of each drug was calculated, see Fig. 2. Each drug exhibits a different profile indicating the complex nature of the interactions between drug and individual media components. The components with the lowest overall influence on solubility are salt (1 significant result from 13 drugs), buffer and monoglyceride (2 from 13), and pancreatin (3 from 13). The components with the biggest overall influence on solubility are bile salt (12 significant results from 13 drugs) followed by pH, oleate and lecithin (10 from 13). This is comparable to the published fasted DoE where pancreatin was the component with the least number of significant influences (1 from 12 drugs) followed by salt (5 from 12 drugs) (Khadra et al., 2015). The mean of the absolute standardised effect value grouped for acidic, basic and neutral drugs is presented in Fig. 3, this provides information on a factor's overall magnitude of influence but masks the effect direction if it is to increase or decrease solubility.

For acidic drugs the component with the biggest magnitude of effect is pH, which is identical to the reported fasted DoE (Khadra et al., 2015) but the value is reduced from ninety to around fifteen. This may be related to the increased concentration and therefore solubilising capacity of the “surfactant” components present in this system. For all drugs the effect is positive, which is identical to the previously reported influence of HIF pH on the solubility of acidic drugs (Clarysse et al., 2009) where for indomethacin this accounted for around 90% of the measured solubility effect. The effects of oleate, bile salt and lecithin concentrations are also generally significant (Fig. 3) but the effect is variable between the drugs (Fig. 2). Bile salt positively affects indomethacin, phenytoin and ibuprofen and negatively valsartan, whilst oleate and lecithin only positively affects indomethacin, zafirlukast and phenytoin. The interaction of bile salt components such as cholic acid with indomethacin has been shown to occur through the hydrophobic domains of both molecules to form the core of a mixed micelle (Prakash et al., 2012). The positive influence of bile salt and phospholipid on indomethacin solubility in HIF has also been previously reported, accounting for approximately 10% of the solubility effect (Clarysse et al., 2009). The remaining components have no significant impact on acidic drug solubility with the exception of a single result for pancreatin on phenytoin.

For basic drugs the influence of pH is not as dominant when compared to the acidic and a more complicated pattern is evident (see Fig. 2, Fig. 3) with pH, oleate and bile salt exhibiting similar effect values with lecithin just significant. In the majority of examples (eight out of twelve, Fig. 2) oleate, bile salt and lecithin exhibit a positive effect on solubility indicating the importance of the surfactant components with some notable negative solubility effects of oleate and bile salt with bromocriptine and lecithin with aprepitant. Monoglyceride, salt, buffer and pancreatin do not influence basic drug solubility with only two out of a possible twenty producing significant effects, pancreatin with aprepitant and carvedilol with buffer.

For neutral drugs, the number of significant factors is even greater with oleate and bile salt showing dominant effects, followed by lecithin and pH with monoglyceride, buffer and pancreatin registering average values that are significant. Oleate, lecithin and bile salt (with the exception of bile salt with fenofibrate) have a positive influence on solubility indicating the importance of the surfactant components for this group of drugs. Surprisingly the effect of monoglyceride is mixed, a result attributable to a positive effect on probucol and fenofibrate offset by a negative effect on felodipine. Whilst the absolute average effect of pH on neutral drugs is significant, Fig. 2 indicates that the solubility effect can be positive or negative. Since in these cases pH cannot influence drug ionisation, this effect is mediated through ionisation of the other media components in a similar manner to that noted in the fasted DoE (Khadra et al., 2015).

The multi-component influences on the solubility of neutral and basic drugs in HIF has been previously reported (Clarysse et al., 2009) and these results are also in agreement with the fasted DoE findings (Khadra et al., 2015) for these drug categories. This emphasises the importance of the solubilising capacity of the media (Ilardia-Arana et al., 2006) and the influence of pH on the solubility of neutral drugs in these systems (Pedersen et al., 2000) acting through ionisation of the solubilising components. It is interesting to note that mono-glyceride, which is included in fed simulated media (Jantratid et al., 2008b, Kleberg et al., 2010) only influenced the solubility of two neutral drugs, fenofibrate and probucol, a result that is similar to literature reports (Kleberg et al., 2010) of oleic acid and monoolein, increasing the solubility of fenofibrate and cinnarizine with limited influence on griseofulvin or danazol.

### 3.3 Solubility influence of DoE factor combinations

**Fig. 5:**
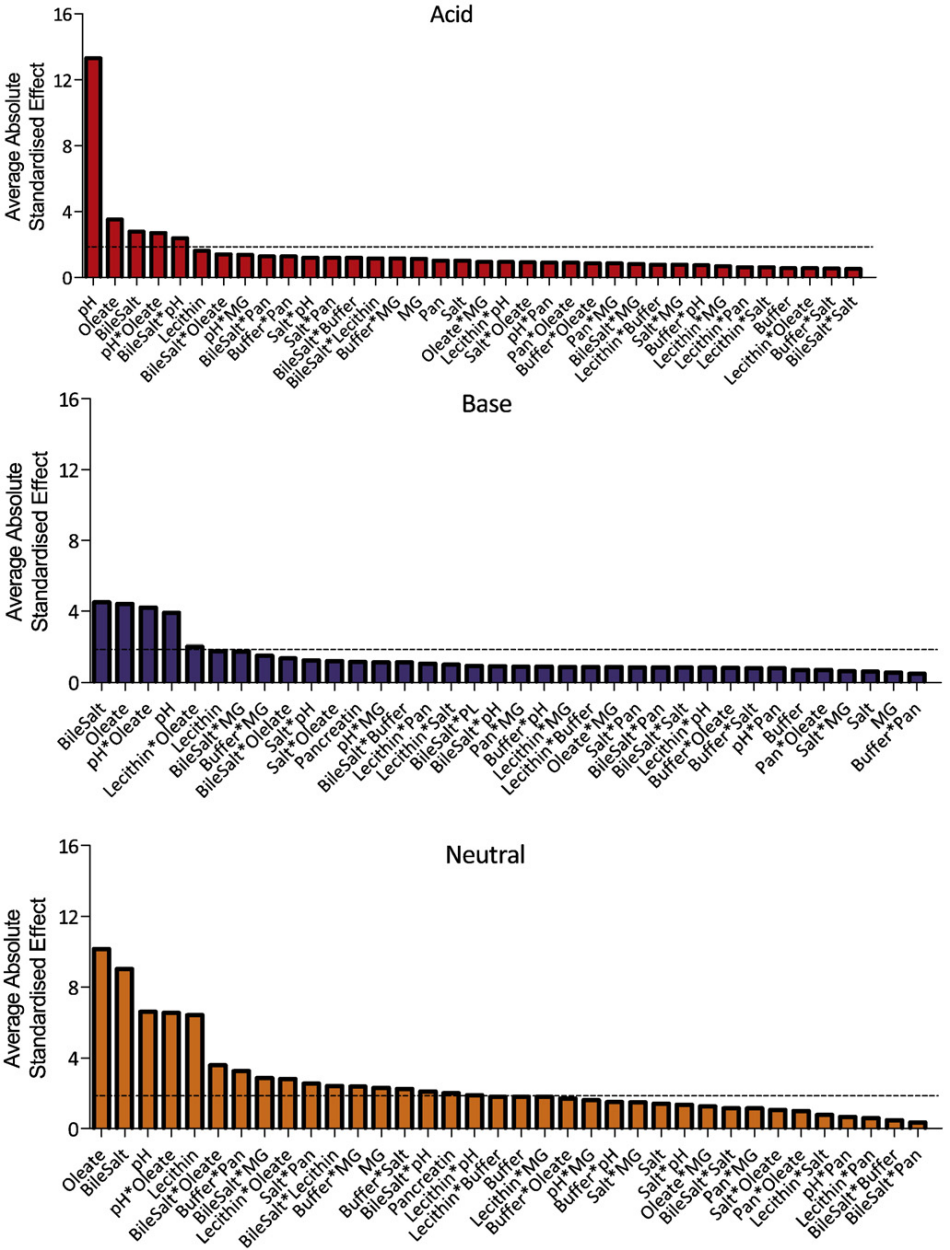
Average absolute standardised effect values for factors and factor interactions grouped by drug category. Average value of the absolute standardised effect for each factor and factor interaction grouped by drug category, note that this removes direction of effect information. Horizontal hatched black line indicates statistical significance (*P* < 0.05).

The DoE provides information on the standardised effect values of interactions between factors on drug solubility and the results are presented in Fig. 4a, Fig. 4b with average absolute values in Fig. 5. Where an interaction occurs it generally has a lower standardised effect than one of the single factors on its own and there are a greater number of significant interactions for the neutral compounds than either acids or bases, see Fig. 5. Interactions have been separated into two groups based on the statistical significance of the average absolute standardised effect value with the neutral compound interactions employed since it is the largest set. A comparison with factor interactions in the fasted DoE is possible but should be treated with caution since several interactions were confounded in these published results (Khadra et al., 2015) and the statistical methods employed are different.

#### 3.3.1 Solubility influence of statistically significant DoE factor combinations

For acidic compounds the only two significant interactions involve pH with either oleate or bile salt, whose pKa's are respectively 5.0 and between 4.5 and 6.5 (Holm et al., 2013). For aqueous fatty acid systems for example it is reported that pH 7 represents a phase change boundary (Cistola et al., 1988) and the aggregation and surface tension properties of bile salts are also pH dependent (Fernández-Leyes et al., 2008). A significant interaction between pH and either oleate or bile salt is therefore to be expected since their ionisation will vary in the DoE pH range and the results indicate that this influences solubilisation. Based on literature results this is probably also linked to changes in the systems phase behaviour (Elvang et al., 2016) however it is interesting that the DoE does not register a significant interaction between bile salt and oleate for the acidic drugs. No other factor combinations produced a statistically significant average value but for individual drugs statistically significant events are present. The interaction between bile salt and lecithin for example is not on average significant however a significant effect is noted for valsartan (Fig. 4a) but no other drugs. It has been previously reported that the combination of bile salt and lecithin in HIF influenced the solublity of indomethacin (Clarysse et al., 2009) at around one tenth of the magnitude of the pH effect. In this study the interaction between these factors on indomethacin solubility was positive at around 1 but not statistically significant (Fig. 4a) with the effect of pH measured at 27, a ratio that is similar to the reported HIF result. The two significant interactions are identical to those reported for the fasted DoE (Khadra et al., 2015) for acids but the significant interaction between buffer and pH noted in the fasted setting was not replicated in the fed.

For basic drugs the average standardised effect values for the interactions between pH and oleate and lecithin and oleate are significant, and similar comments to those for the acids above with respect to pH apply. However, since lecithin ionisation is not influenced by pH this may represent a three way (or greater) interaction with pH influencing an ionisable component, for example oleate, which in turn interacts with the lecithin. For practical reasons related to the number of experiments the DoE is not powered to examine this type of relationship. There are occasional significant interactions for other factors with individual drugs, for example a negative solubility effect of lecithin and oleate with aprepitant and a positive effect of bile salt and monoglyceride with carvedilol. However, there is no obvious consistent pattern with these interactions, which indicates that they may arise through the drugs structural and physicochemical properties (Kleberg et al., 2010). For nifedipine and ketoconazole pH, fatty acids and the combination of bile salt and phospholipids have been shown to influence solubility (Clarysse et al., 2009) but interactions between the factors was not determined. Similar to the acidic compounds the number of significant interactions in the fed DoE is fewer than the fasted DoE (Khadra et al., 2015) with only pH and oleate significant in both cases. None of the other twenty six possible interactions were significant for the basic drugs.

For neutral drugs the pattern of factor interactions is more complicated with fifteen significant results based on the average absolute standardised effect values, providing in some cases (for example pH with oleate) effect values comparable with the single factor result, see Fig. 4a, Fig. 5. The most frequent factors involved in significant interactions are buffer and lecithin in five interactions each followed by oleate, bile salt, monoglyceride and pH in four and salt and pancreatin in two. The average standardised effect value removes information on the direction of the effect and Fig. 4a emphasises the complex nature of the solubility pattern. For example, the interaction between bile salt and oleate or lecithin results in a solubility increase for all the neutral drugs, which is in line with literature results for these components (Soderlind et al., 2010). However, pH and oleate interact to increase the solubility of felodipine and fenofibrate but decrease the solubility of itraconazole and probucol, indicating that in some cases the drug's molecular properties are influencing the outcome. Generally, the interactions between the surfactant components (bile salt, lecithin, oleate and monoglyceride) act to increase solubility, with a few exceptions, whilst those involving buffer and salt tend to decrease solubility, with the effect of the remaining interactions being variable. This pattern is similar to the results from the fasted media DoE (Khadra et al., 2015) where the bile salt, lecithin interaction had a uniformly positive effect on solubility whilst pH and oleate was variable. Overall these results indicate that for neutral drugs solubility in simulated fed media is a complex phenomenon that will be dependent on the ratio of multiple components and the interactions between them (Birru et al., 2014, Kleberg et al., 2010).

#### 3.3.2 Solubility influence of statistically non-significant DoE factor combinations

The factor interactions where the average absolute standardised effect value was not statistically significant for neutral drugs are presented in Fig. 4b. Note that this is based on the neutral drugs where thirteen of the possible twenty eight interactions are non-significant a value that is lower than the acidic or basic drugs where twenty six of twenty eight interactions are non-significant. Based on the neutral drugs the most frequently non-significant factor interactions at five times each are pancreatin and salt a similar pattern to the fasted DoE (Khadra et al., 2015). The remaining factors occur with roughly equal frequency and only one interaction between surfactant components, oleate with monoglyceride, is present. However, it should be noted that even though the interaction ranking is based on the absolute average standardised effect value, significant effects on individual drugs are still present, for example bile salt with buffer or pancreatin has a positive effect on the solubility of indomethacin.

### 3.4 Combined factors and factor interactions

The DoE consisted of eight factors and a possible twenty eight interactions between the factors and the ranked average absolute standardised effect value for each group of drugs is presented in Fig. 5. This illustrates the difference in the impact of the various factors and factor interactions for the three drug categories. Acidic drug solubility is influenced by pH, oleate and bile salt or by interactions between these factors, with the influence of pH dominant. The remaining five factors and twenty six interactions are not significant. This is similar to the fasted DoE (Khadra et al., 2015) with the exception that buffer and lecithin were also significant. For basic drugs pH, oleate, bile salt and lecithin as individual factors are significant along with interactions between pH and oleate and lecithin and oleate. The remaining four factors and twenty six interactions are not significant. In a similar manner to the acids this is a smaller number when compared to the fasted DoE where buffer and salt as single factors along with interactions between bile salt and oleate, lecithin and salt, bile salt and buffer, lecithin and pH and salt and pH were significant. In both acids and bases this difference, as discussed above, may represent a dominance of the surfactant based components due to their higher concentration in the fed state. For neutral compounds a more complicated picture is evident with only salt as single factor not significant and fifteen of the possible twenty six interactions significant. This is similar to the fasted DoE where only pancreatin was non-significant as a single factor. The interactions which are no longer significant when comparing the fasted and fed DoEs are salt and pH, lecithin and salt, oleate and salt and bile salt and buffer, again this may represent a dominance of the surfactant based components due to their higher concentration in the fed state. Although it should be noted that the handling of factor interactions differs in each DoEs. Overall the results indicate that the solubilisation of neutral compounds in fed simulated intestinal media is a complex relationship contributed to by the individual factors and numerous interactions (Clarysse et al., 2009, Kleberg et al., 2010).

## 4 Conclusions

The purpose of this study was to determine the feasibility of extending the previous study on fasted simulated intestinal media into fed simulated intestinal media and determine any shifts in performance. The results indicate that utilising a design of experiment technique is feasible for the determination of equilibrium solubility and provides data that is comparable in magnitude and variability to literature results in both HIF (Augustijns et al., 2014) and fed simulated intestinal media (Clarysse et al., 2011). The information provides greater detail on the interactions than previous approaches which have varied complete media or single components and has greater versatility than sampled HIF. The technique provides a measure of the average solubility and variability along with the importance of the media factors and factor interactions which influence solubility.

The three drug groups acidic, basic and neutral display different profiles with respect to the most significant factor and factors interactions influencing solubility. For acidic drugs pH is dominant although with a reduced margin and similar although fewer in number single factors and interactions are present to those in the fasted DoE. Basic drugs follow the same pattern in comparison to the fasted DoE as acidic drugs and indicates that the surfactant components, especially oleate, bile salt and lecithin are dominant over other factors (salt, buffer, pancreatin) due to their higher concentration in fed simulated media. For neutral drugs a more complex picture is evident with seven out of eight single factors significant plus over half of the possible interactions, indicating that for these drugs solubilisation in fed simulated media is a complex interplay. The DoE can also determine individual specific interactions between drugs and media factors that do not follow the overall trend within a group. Since pH is the predominant single factor influencing solubility and in vivo pH is influenced by gastrointestinal gas (Fiddiangreen et al., 1982) production and disposition (Mego et al., 2015) the influence of carbon dioxide as a buffering system would be an interesting experimental modification. A number of factors were not significant or only had low impact for example pancreatin, salt and buffer and in future studies these could be removed to limit the number of experiments required or reduce the factors to increase detail. A judicious approach would be required since pancreatin would be important for the performance of lipid based formulations where digestion is important (Williams et al., 2012).

This DoE with ninety two experiments and the fasted with sixty six is over one hundred and fifty experiments somewhat larger than the original goal of developing a technique suitable for 96 well plates (Khadra et al., 2015). In future studies combining fed and fasted conditions along with modification of the DoE to only examine biorelevant combinations will be required to meet this goal of describing a drug's gastrointestinal solubility envelope in a single DoE. If this can be realised then the measured maximum, minimum and average solubilities could be applied to the Developability Classification System (Butler and Dressman, 2010) to provide a classification range. In addition measured intrinsic dissolution rates using the maximum, minimum and average solubility simulated media could be applied to refine PBPK models of drug absorption (Kostewicz et al., 2014) permitting determination of gastrointestinal content changes either through food intake or resulting from transfer through the tract (Clarysse et al., 2009).

## Figures and Tables

**Fig. 4a:**
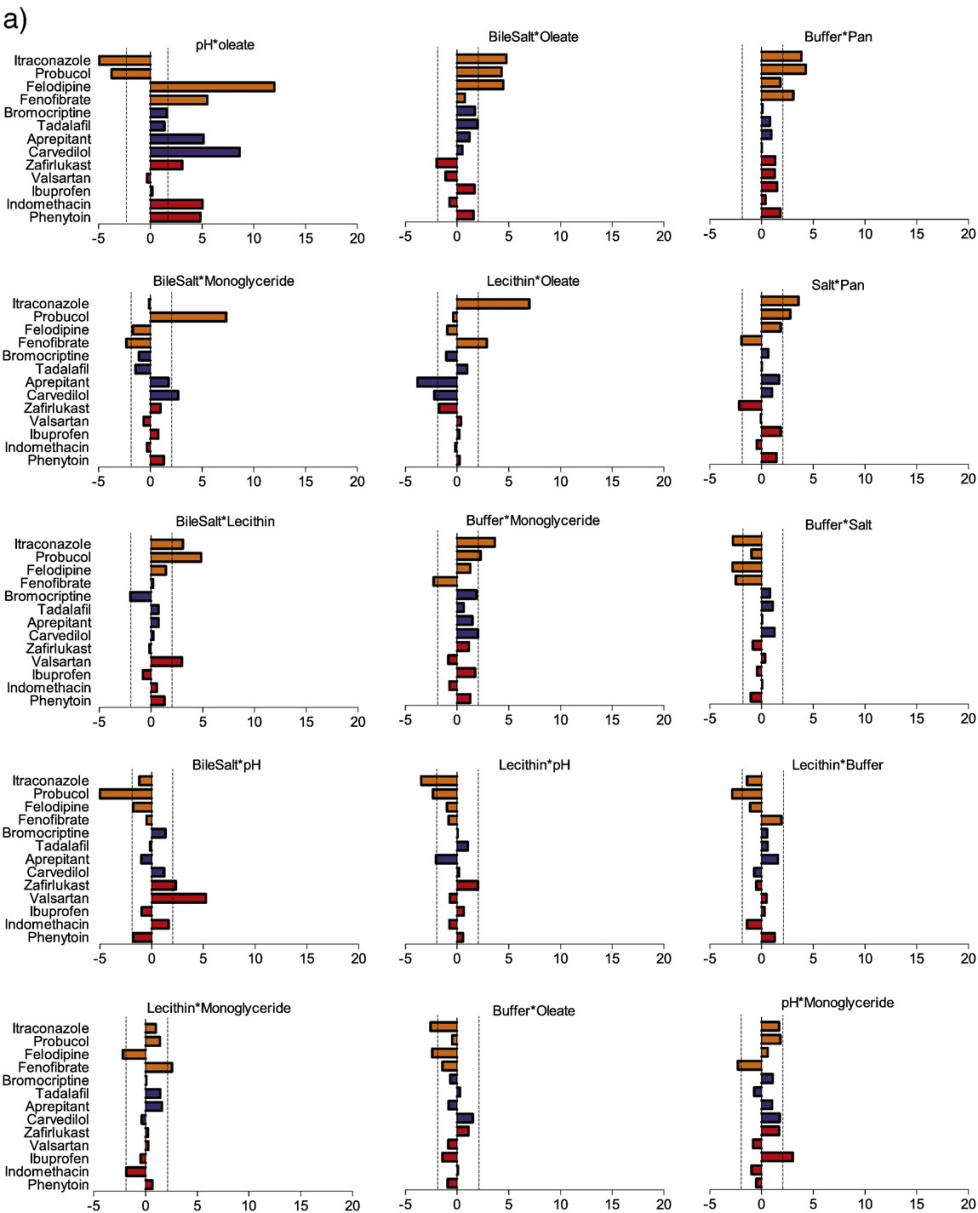
Standardised effect values for doe factor interactions on equilibrium solubility. DoE standardised effect values (x-axis) for individual factors (as listed in figure titles) on equilibrium solubility. Vertical hatched black lines indicate statistical significance (*P* < 0.05), bar direction indicates direction of effect, to the right of 0 on x-axis is a positive effect on solubility, bar length indicates the magnitude of the effect. Interactions which provide a statistically significant average absolute standardised effect value included. Abbreviations; Pan - pancreatin; Mono – monoglyceride. Acidic drugs red coloured bars; Basic blue coloured bars; Neutral yellow coloured bars.

**Fig. 4b:**
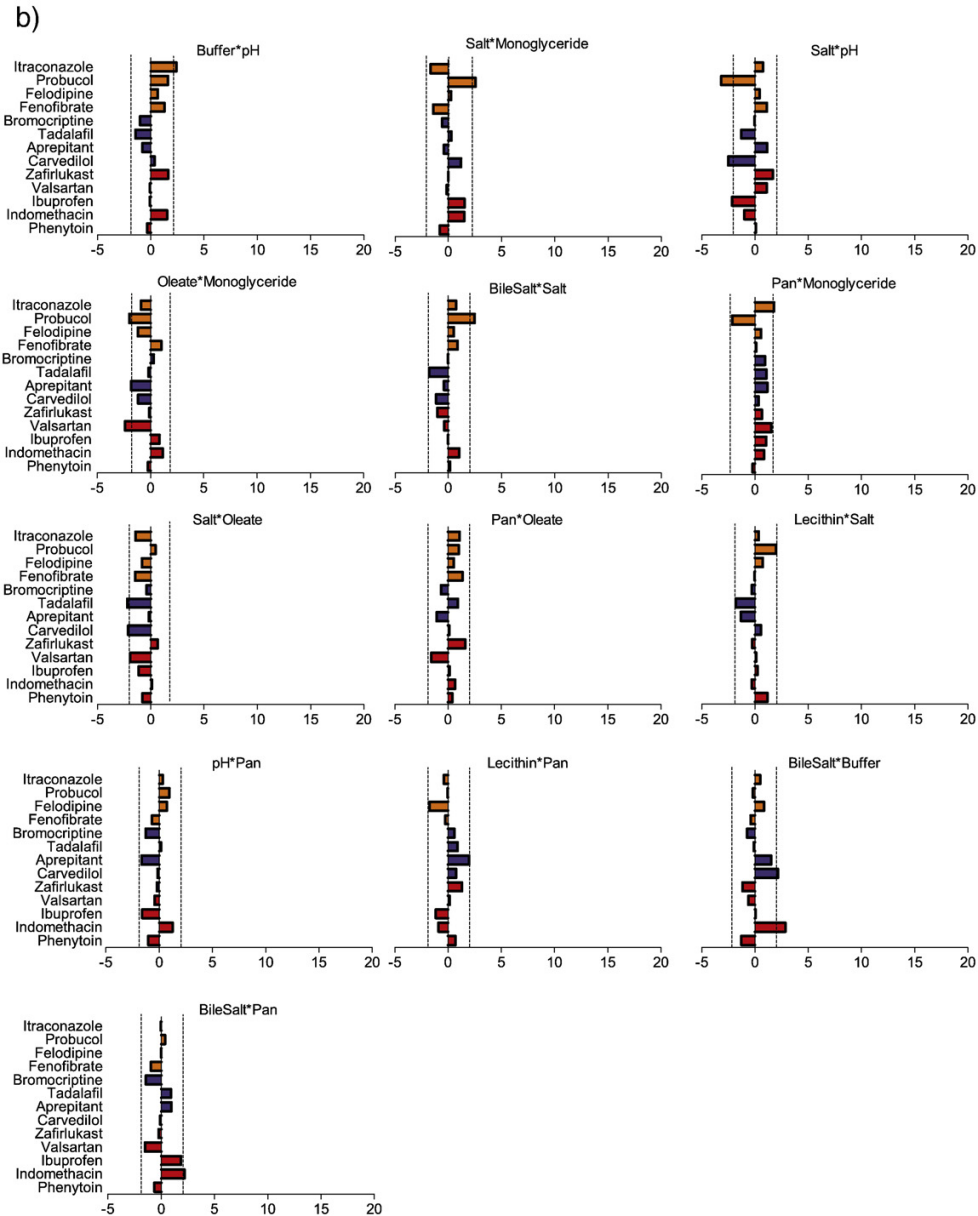
Standardised effect values for doe factor interactions on equilibrium solubility. DoE standardised effect values (x-axis) for individual factors (as listed in figure titles) on equilibrium solubility. Vertical hatched black lines indicate statistical significance (*P* < 0.05), bar direction indicates direction of effect, to the right of 0 on x-axis is a positive effect on solubility, bar length indicates the magnitude of the effect. Interactions which do not provide a statistically significant average absolute standardised effect value included. Acidic drugs red coloured bars; Basic blue coloured bars; Neutral yellow coloured bars.

**Table 1:** Composition of literature fed simulated intestinal media (FeSSIF).

	Dressman et al., 1998 (FeSSIF)	Galia et al., 1998 (FeSSIF)	Vertzoni et al., 2004	Jantratid et al., 2008a (FeSSIF-V2)	Kleberg et al., 2010
pH	5	5	5	5.8	6.5
Buffer	Acetate	Acetate	Citrate	Maleate	Maleate
Sodium taurocholate	15 mM	15 mM	15 mM	10 mM	5–20 mM
Lecithin	4 mM	3.75 mM	3.75 mM	2 mM	1.25–5 mM
BS/PL	3.75	4	4	5	4
Salt	0.19 M (KCl)	0.20 M (KCl)			
Sodium oleate	–	–	–	0.8 mM	0–45 mM
Mono-oleate	–	–	–	5 mM	0–10 mM

**Table 2:** Composition and concentration levels employed in design of experiment for fed simulated intestinal media.

Parameter	Substance	Lower limit	Upper limit
Bile salt (mM)	Sodium TC	3.6	24
Lecithin (mM)	Egg PL	0.5	4.8
Buffer (mM)	Maleic acid	28.6	58.09
Salt (mM)	NaCl	125	203
pH	NaOH/HCl	5	7
Enzyme (U/ml)	Pancreatin	100	150
Fatty acid (mM)	Sodium oleate	0.8	52
Monoglyceride	Glycerylmonooleate	1	6.5

TC: taurocholate, PL: phosphatidylcholine.
